# The Same All the Time

**Published:** 1879-02

**Authors:** 


### THE SAME ALL THE TIME.

"Choose your friends of such as are the same all the time," was a sententious pulpit utterance, during the great awakening. What a regiment of monosyllables! and yet conveying a meaning which it would require a whole book to express. A real good old Anglo-Saxon phrase, is it. The sentiment was uttered in reference to the propriety, and greater safety of the newly-made members of the Church, taking as their patterns and companions, not the men who had so suddenly become zealous—so much so, as to seem to be carrying all before them; but rather the old stagers, who did not seem so very fervent; who were neither very fast, nor very slow; neither running at any time, nor stopping at any time; were rather going at a steady pace, from one year's end to another's; who were all the time the same humble, steady, earnest, reliable workers in the Church; never a flash, never an icicle.

Young man! there is good counsel in this for you, in life's beginning. It is the steady labor which accomplishes the most work. The impulsive are uncertain. Without steadiness of purpose, few succeed in life. There are fast men, who, for a season, distance everybody, only to die prematurely, or in poverty, and that not always an honorable one. In the early morning, all vigorous and fresh, the pig shot away from the tortoise, and soon left it out of sight; but, long before the close of the day, was passed, lying exhausted—and the turtle waddled along to victory, apparently as ready for a new race as it was in the morning. In business, then, as well as in religion, for your exemplars, your associates, and your friends, choose men who, in a high sense, "are the same all the time."

